# Chromatin Remodeling BAF (SWI/SNF) Complexes in Neural Development and Disorders

**DOI:** 10.3389/fnmol.2017.00243

**Published:** 2017-08-03

**Authors:** Godwin Sokpor, Yuanbin Xie, Joachim Rosenbusch, Tran Tuoc

**Affiliations:** ^1^Institute of Neuroanatomy, University Medical Center, Georg-August-University Goettingen Goettingen, Germany; ^2^DFG Center for Nanoscale Microscopy and Molecular Physiology of the Brain Goettingen, Germany

**Keywords:** epigenetic regulation, chromatin remodeling, BAF (mSWI/SNF) complex, neural development, neurodevelopmental disorder

## Abstract

The ATP-dependent BRG1/BRM associated factor (BAF) chromatin remodeling complexes are crucial in regulating gene expression by controlling chromatin dynamics. Over the last decade, it has become increasingly clear that during neural development in mammals, distinct ontogenetic stage-specific BAF complexes derived from combinatorial assembly of their subunits are formed in neural progenitors and post-mitotic neural cells. Proper functioning of the BAF complexes plays critical roles in neural development, including the establishment and maintenance of neural fates and functionality. Indeed, recent human exome sequencing and genome-wide association studies have revealed that mutations in BAF complex subunits are linked to neurodevelopmental disorders such as Coffin-Siris syndrome, Nicolaides-Baraitser syndrome, Kleefstra's syndrome spectrum, Hirschsprung's disease, autism spectrum disorder, and schizophrenia. In this review, we focus on the latest insights into the functions of BAF complexes during neural development and the plausible mechanistic basis of how mutations in known BAF subunits are associated with certain neurodevelopmental disorders.

## Introduction

The developmental processes that lead to formation of the central nervous system (CNS) are intricately regulated to ensure proper cellular architecture and functionality. Mammalian neural structures are functionally made up of neural cells that develop under strict molecular and cellular instructions to confer cell subtype differentiation. During CNS development, cell fate acquisition is initiated by the decline in neural stem cell self-renewal and amplification potentials to yield fate-committed progenitors that ultimately undergo neurogenic or gliogenic differentiation as reviewed in Guillemot (2007), Kriegstein and Alvarez-Buylla (2009), Taverna et al. (2014) and Tuoc et al. (2014). Highly ordered gene expression programs regulate the establishment of various cell fate status during neural development. Many factors, including epigenetic and chromatin regulators act in concert to determine and sustain cell-specific transcriptional programs. The establishment of specific epigenetic landscapes provides strong evidence that link chromatin state with gene expression profiles. Thus, regions of DNA compaction (heterochromatin) are transcription-repressed sites, whereas less compact or open regions (euchromatin) have active transcriptional activity (Hirabayashi and Gotoh, 2010; Juliandi et al., 2010; Coskun et al., 2012; Ronan et al., 2013; Narayanan and Tuoc, 2014; Watson and Tsai, 2016; Yao et al., 2016). Chromatin regulators are capable of reordering chromatin state (heterochromatin/euchromatin) and hence are critical determinants of access to genomic loci by the transcriptional machinery (Hirabayashi and Gotoh, 2010; Juliandi et al., 2010; Coskun et al., 2012; Ronan et al., 2013; Narayanan and Tuoc, 2014; Watson and Tsai, 2016; Yao et al., 2016). Consequently, a coordinated deployment of chromatin regulators, along with the recruitment of transcription factors, are essential for establishing gene expression patterns in response to various cellular cues and physiological states in health and disease.

During development, signals within and outside cells cause genome-wide chromatin dynamics that correspond with instructive gene expression patterns for cell lineage specifications. For instance, during cortical neurogenesis, various chromatin remodeling machinery are actively involved in directing the expression of numerous neurogenic gene targets, leading to progenitor proliferation and/or differentiation (Juliandi et al., 2010; Ronan et al., 2013; Narayanan and Tuoc, 2014).

Chromatin structure can be regulated at various levels, including covalent modification (e.g., methylation, acetylation, ubiquitination, and phosphorylation) of DNA, RNA, histones, and remodeling of nucleosome by ATP—dependent chromatin remodeling factors, as well as activities of non-coding RNAs (Strahl and Allis, 2000; Goldberg et al., 2007; Kouzarides, 2007). Epigenetic factors act together to instruct transcriptional regulation through direct interaction with transcription factors at genomic loci. ATP-dependent chromatin factors are particularly known powerful regulators of chromatin state and DNA accessibility to transcription factors that are critical for transcriptional regulation and gene expression outcomes (Ronan et al., 2013; Narayanan and Tuoc, 2014).

In our review, we will concentrate on the multi-subunit mammalian SWItch/Sucrose Non-Fermentable (mSWI/SNF), also known as BRG1/BRM associated factor (BAF) chromatin modifier family that are capable of utilizing ATP energy to alter nucleosomal units in chromatin structure, culminating in heterochromatin-euchromatin state inter-convertibility. The mSWI/SNF complexes play central roles in epigenetic regulatory mechanisms that impact on developmental processes. Emerging findings implicate the mSWI/SNF complex in orchestrating rate-limiting epigenetic regulation of various pre- and post-natal neural developmental events (Matsumoto et al., 2006; Lessard et al., 2007; Wu et al., 2007; Weider et al., 2012; Li et al., 2013; Ninkovic et al., 2013; Tuoc et al., 2013a,b, 2016; Vogel-Ciernia et al., 2013; Yu et al., 2013; Narayanan and Tuoc, 2014; Bischof et al., 2015; Narayanan et al., 2015; Wiegreffe et al., 2015; Bachmann et al., 2016; Nguyen et al., 2016). However, similar to other biological systems, abnormal chromatin regulation can sometimes occur. Chromatin remodelers are thus amenable to alterations and mutations that can lead to aberrant chromatin modifications capable of engendering developmental disorders and homeostatic disturbances during neural development. Of special interest, due to their prominent neurodevelopmental functions, misregulation or mutations of mSWI/SNF complex subunits cause neurological disorders with syndromic and non-syndromic cognitive dysfunction phenotypes (Ronan et al., 2013). Notably, Coffin-Siris syndrome, Schizophrenia, Nicolaides-Baraitser syndrome, and autism spectrum disorders are characterized human diseases caused by defective mSWI/SNF complexes. As such, the link between mSWI/SNF complexes and neural development in health and disease is increasingly attracting investigative efforts aimed at elucidating the mechanisms involved.

This review focuses on the emerging role of the mSWI/SNF complex as chromatin regulation factor, and its involvement in neurodevelopmental processes such as neural stem cell/precursor generation, proliferation, and neuronal subtype specification, differentiation, and migration. To that end, we will detail proven findings of the functions of the mSWI/SNF complex in various neural perturbations.

## Biochemical characteristics of the mSWI/SNF (BAF) complexes

The mSWI/SNF (BAF) complex was originally identified in yeast (Neigeborn and Carlson, 1984; Wang et al., 1996). The BAF complex comprises at least 15 subunits of ~2 MDa in size, and thus more multimeric than the yeast version (Lessard et al., 2007; Wu et al., 2007). Typically, there is enrichment of BAF complex at promoters (Ho et al., 2009a,b, 2011), and particularly much more variable at (super)enhancers (Bossen et al., 2015; Barutcu et al., 2016; Alver et al., 2017; Wang et al., 2017) of active genes involved in cellular processes such as proliferation, cell identity, differentiation, and motility.

**Figure 1:**
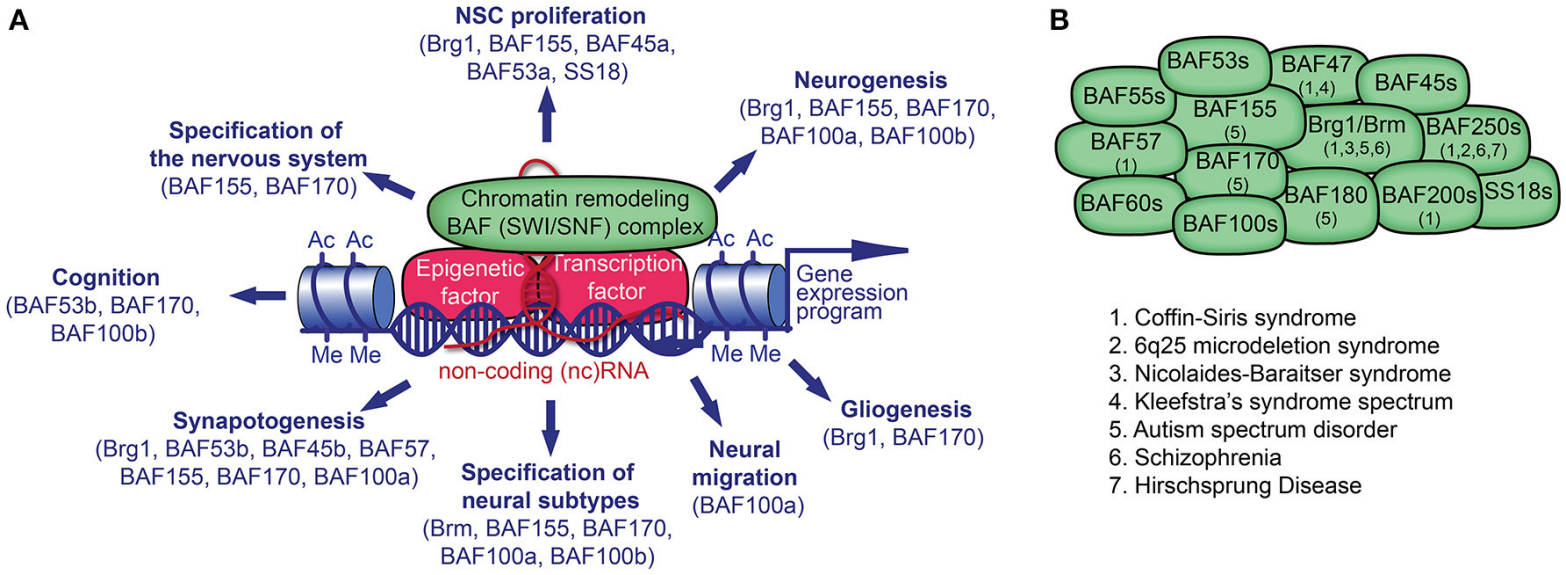
Chromatin remodeling BAF (mSWI/SNF) complex in neural development and disorders. **(A)** The BAF complex, epigenetic factors (including non-coding [nc] RNA), and transcription factors (TF) control gene expression. TFs and ncRNAs bind to specific DNA sequences. The recruitment of BAF complexes and other epigenetic factors on the genome leads to altered epigenetic marks (e.g., histone acetylation, Ac; histone methylation, Me) and chromatin structure in order to activate or repress a specific gene expression program in cell lineages. Many BAF subunits as indicated, regulate distinct processes of neural development. **(B)** The presence of known BAF subunits in different BAF complexes in neural cells is indicated. The mutation of genes encoding for the noted BAF subunits has been reported in various neurological disorders.

The complex is usually made up of exchangeable ATPase core(s), and other core subunits including scaffolding proteins around which the entire complex is assembled. Brahma (BRM) and BRM/SWI2 related gene 1 (BRG1) are the only known two catalytic ATPase subunits out of the 29 possible SWI2/SNF2-like ATPases encoded by the mammalian genome that are incorporated into the BAF complex (Neigeborn and Carlson, 1984; Wang et al., 1996; Lessard et al., 2007; Wu et al., 2007; Kadoch et al., 2013; Narayanan et al., 2015; Bachmann et al., 2016). The core subunits include ubiquitously expressed BAF47, BAF155, and BAF170 (Phelan et al., 1999) (see Table 1 and Figure 1 for a list of BAF subunits). Many core and variant BAF subunits contain DNA- and histone-binding domains such as zinc finger, AT-hook (including high mobility group [HMG] proteins), AT-rich interaction domain (ARID), plant homeodomain (PHD), helicase/SANT-associated (HSA) domain, “switching-defective protein 3 (Swi3), adaptor 2 (Ada2), nuclear receptor co-repressor (N-CoR), transcription factor (TF)IIIB” [SANT] domain, as well as chromo- and bromodomains, which afford transcription factor recruitment specificity, genome targeting, and protein-protein interactions. As such, the BAF complex has an autonomous capacity to specifically regulate gene expression patterns in a cell lineage restricted manner. *In vivo*, the BAF complex is dynamic and exhibits considerable plurality. The existence of several subunit variants and polymorphic sites is believed to make it possible to distinctively assemble hundreds of BAF complexes in a combinative fashion (Lessard et al., 2007; Wu et al., 2007; Kadoch et al., 2013; Bachmann et al., 2016).

**Table 1:** BAF subunits and their role in aspects of neural development.

**BAF subunit**	**Type of mutant**	**Mutants neural lineage**	**Phenotype**	**References**
**NEURAL SPECIFICATION**				
BAF155 BAF170	*FoxG1-Cre;* *BAF155*^*fl*/*fl*^; *BAF170*^*fl*/*fl*^	Telencephalic NSCs	Telencephalon is not specified	Narayanan et al., 2015
BAF155 BAF170	*FoxG1-Cre;* *BAF155*^*fl*/*fl*^; *BAF170*^*fl*/*fl*^	Olfactory epithelium NSCs	Olfactory epithelium is not specified	Bachmann et al., 2016
**NSC PROLIFERATION**				
BRG1	*Nestin-Cre;* *Brg*^*fl*/*fl*^	Telencephalic NSCs	Defect in self-renewal and maintenance of murine NSC	Matsumoto et al., 2006; Lessard et al., 2007; Zhan et al., 2011
	*Wnt1-Cre;* *BRG1*^*fl*/*fl*^	NCCs	Defect in proliferation of NCCs	Li et al., 2013
BAF45a	*BAF45a^*kd*^*	Cortical neural stem/progenitor cells	Impaired neural stem/progenitor proliferation	Lessard et al., 2007
	*BAF45a^*OE*^*	Cerebellar progenitor cells	Extended proliferative phase of cortical neural stem/progenitor and cerebellar precursor cells	Lessard et al., 2007
BAF53a	*BAF53a^*kd*^*	Cortical neural stem/progenitor cells	Impaired neural stem/progenitor proliferation	Lessard et al., 2007
BAF155	*BAF155^−/−^*	Null mutation	Abnormal proliferation and differentiation in heterozygotes	Kim et al., 2001
	*BAF155^*fl*/*fl*^; FoxG1-Cre*	Olfactory epithelium NSCs (oNSCs)	Defective proliferation of oNSCs	Bachmann et al., 2016
SS18	*Ss18^−/−^,Ss18^*kd*^*	Null mutation; SS18 KD	Defect in closure of neural tube, NSC proliferation, dendritic outgrowth	de Bruijn et al., 2006; Staahl et al., 2013
**EMBRYONIC NEUROGENESIS**				
BAF170	*BAF170*^*fl*/*fl*^; *CMV-Cre;* *Cre-eGFP*	Cortical progenitors	Aberrant expansion in IP pool; increased indirect neurogenesis	Tuoc et al., 2013b
	*BAF170^*OE*^*	Cortical progenitors	Depleted upper cortical layer neurons	Tuoc et al., 2013b
BAF170	*Emx1-Cre; BAF170^*fl*/*fl*^*	Cortical progenitors	Increased genesis of IPs, enhanced cortical volume, surface area and thickness	Tuoc et al., 2013a
	*BAF170^*OE*^*	Cortical progenitors	Decreased genesis of IPs, diminished cortical volume, surface area and thickness	Tuoc et al., 2013a
**ADULT NEUROGENESIS**				
Ctip2/Bcl11b	*Ctip2^−/−^*	Targeted deletion of *Ctip2* gene	Medium spiny neurons hippocampal neurogenesis	Simon et al., 2012
BRG1	*Glast-Cre;* *BRG1*^*fl*/*fl*^	BRG1 deletion in aNSC and astrocytes	Defect in neurogenesis of aNSC in SVZ	Matsumoto et al., 2006; Ninkovic et al., 2013
BAF170	*hGFAP-Cre or* *Nestin-CreER;* *BAF170*^*fl*/*fl*^	BAF170 deletion in aNSC in DG	Defect in neurogenesis of aNSC in DG	Tuoc et al., 2016
**NEURONAL SUBTYPE SPECIFICATION**				
Ctip2/Bcl11b	*Ctip2^−/−^*	Targeted deletion of *Ctip2* gene	Specification of subcerebral projection neurons	Arlotta et al., 2005, 2008
Ctip1/Bcl11a	*Ctip1^*fl*/*fl*^;* *Emx1-Cre*; *Nex1-Cre*	Cortical progenitors and post-mitotic neurons	Specification of subcerebral projection neurons; Reduced Tbr1 and Ctip2 expression; disrupted cortical projection neuron pathfinding	Woodworth et al., 2016
**NEURONAL MIGRATION**				
Ctip1 (Bcl11a)	*Bcl11a^*fl*/*fl*^*; *Emx1-Cre*; *Nex-Cre*	Cortical neural progenitors; post-mitotic neurons	Impaired radial migration due to defective multipolar to bipolar morphology, Cell accumulation in IZ transition; dysplasia of upper cortical layers	Wiegreffe et al., 2015
**NEURONAL MATURATION**				
Crest/Ss18l1	*Crest^−/−^*	Null mutation	Defects in dendrite development	Aizawa et al., 2004; Qiu and Ghosh, 2008
Ctip1/Bcl11a	*Brn4-Cre;* *Ctip1*^*fl*/*fl*^		Defect in neuronal morphogenesis in spinal cord	Kuo et al., 2010a,b; Estruch et al., 2012; John et al., 2012
Ctip2/Bcl11b	*Ctip2^−/−^*	Targeted deletion of *Ctip2* gene	Defect in the differentiation of vomeronasal sensory neurons	Enomoto et al., 2011
BAF155	*BAF155^*fl*/*fl*^;* *FoxG1-Cre*	ORN	Impaired maturation and axonogensis of ORNs	Bachmann et al., 2016
BAF170	*BAF170^*fl*/*fl*^*; *FoxG1-Cre*	ORN	Impaired maturation and axonogensis of ORNs	Bachmann et al., 2016
**OLIGODENDROGENESIS**				
BRG1	*BRG1^*fl*/*fl*^;* *Olig1Cre^+/−^*	BRG1 deletion from OPCs	Impairment of OL differentiation and maturation; Myelin-deficient phenotypes: generalized tremors, hind limb paralysis and seizures	Yu et al., 2013
**ASTROGENESIS**				
BRG1	*Glast-Cre;* *BRG1^*fl*/*fl*^*	BRG1 deletion in aNSC and astrocytes	Defect in astrogenesis	Matsumoto et al., 2006; Ninkovic et al., 2013
	*BRG1^*fl*/*fl*^;* *Nestin-Cre*	Glial precursors	Perturbed astrocytic differentiation	Matsumoto et al., 2006
BAF170	*hGFAP-Cre; or* *Nestin-CreER;* *BAF170^*fl*/*fl*^*	BAF170 deletion in aNSC in DG.	Premature differentiation of aNSCs to astrocytes in DG	Tuoc et al., 2016
**SCHWANN CELL GENERATION**				
BRG1	*Dhh-Cre;* *BRG1^*fl*/*fl*^*	BRG1 deletion in Schwann cells	Defect in differentiation and myelination of Schwann cells	Weider et al., 2012; Limpert et al., 2013
**LEARNING AND MEMORY**				
BAF53b	*BAF53b^−/+^*	Hippocampal post-mitotic neurons	Defective synaptic plasticity; Long term memory formation impairment	Vogel-Ciernia et al., 2013
	*Camk2α promoter;* *BAF53bΔHD*	Expression of dominant negative BAF53b in forebrain excitatory neu-rons	Abnormal spine structure and function; decline in cognitive functions	Vogel-Ciernia et al., 2013
	*BAF53b^−/−^*	Null mutation	Defective dendritic outgrowth and synapse formation	Wu et al., 2007
Ctip2	*Bcl11b^*fl*/*fl*^;* *Emx1-Cre*	Post-mitotic granule neuron in SGZ	Reduced size of DG; impaired spatial learning and memory; defective granule cell differentiation	Simon et al., 2012
BAF170	*hGFAP-Cre* *NEST-CreER*	RGL cells	Impaired adaptive behavior	Tuoc et al., 2016

*(a)/(o)NSC, (adult)/(olfactory) neural stem cell; IPs, intermediate progenitors; NCC, Neural Crest Cell; OL, oligodendrocyte; OPC, oligodendrocyte precursor cells; oRN, olfactory receptor neurons; SVZ, subventricular zone; KD, knock-down*.

Mechanistically, a BAF complex remodels chromatin using its ATPase core subunits to hydrolyze ATP and hence generate energy for nucleosomal unwrapping, mobilization, ejection, or histone dimer exchange (Cairns, 1998; Phelan et al., 1999; Whitehouse et al., 1999; Saha et al., 2002; Gutiérrez et al., 2007; Tang et al., 2010). This process relaxes condensed chromatin (heterochromatin) to increase accessibility of transcription factor binding to activate gene expression (Hara and Sancar, 2002; Gong et al., 2006; Ho et al., 2009a; Hu et al., 2011; Tolstorukov et al., 2013). The converse holds true whereby BAF complex disengagement leads to polycomb repressor complex-driven re-establishment of heterochromatin signatures associated with gene repression (Boyer et al., 2005; Ho et al., 2011; Kadoch et al., 2017).

Various studies consolidate the notion that BAF complex subunits are widely expressed, and the specific combinatorial assemblies and subunit switching contribute to the formation of cell lineage specific BAF complexes capable of instructing specific cell fates *In vivo* (Lessard et al., 2007; Wu et al., 2007; Tuoc et al., 2013a; Bachmann et al., 2016). For instance, the epigenetic landscape in embryonic stem (ES) cells include specific BAF complex needed for maintenance of their proliferative (self-renewal) capacity and pluripotency. The ES cell BAF (esBAF) complex so established is characteristically composed of BRG1, BAF60a/b, BAF155, and BAF250a, and excluding their site-specific polymorphic variants, which are BRM, BAF60c, BAF170, and BAF250b, respectively (Kaeser et al., 2008; Ho et al., 2009a; Kidder et al., 2009). Similarly, during neural development, the BAF complex participates in stereotypic patterns that lead to formation of neural tissue, thus indicating some form of customization of BAF complex therein. After receiving the appropriate stimuli, ES cells differentiate into neural stem cells (NSCs). Accordingly, reconstitution of the esBAF produces neuronal progenitor BAF (npBAF) complex, which subsequently switch to neuron-specific BAF (nBAF) complex during differentiation (Lessard et al., 2007; Wu et al., 2007; Bachmann et al., 2016). The significance of both npBAF and nBAF complexes in neural development is discussed further in the next two sections.

## Function of BAF complex(es) in development of the nervous system

The ubiquitous expression of the chromatin remodeling BAF complex reflects its extensive involvement in controlling embryogenesis (Smith-Roe and Bultman, 2013; Alexander et al., 2015; Nguyen et al., 2016). The BAF complex has been shown in many studies to be required in the maintenance of neural development and the establishment of fully functional nervous system. Such studies explain the critical role of BAF complex in altering chromatin state to mainly influence gene expression patterns that greatly impact on neurodevelopmental events, such as specification of brain structures, neurogenesis (cell proliferation, fate specification, differentiation), cell migration and maturation, and functional integration of neurons. Plausible modulatory mechanisms that may explain how BAF complex particularly influences neurodevelopmental events among other biological processes likely include its spatiotemporal antagonistic interaction with the polycomb complex (Ho et al., 2011; Kadoch et al., 2017) and microRNA-mediated re-composition of its subunit(s) in neural tissues during development (Yoo and Crabtree, 2009). Interestingly both BAF and polycomb complexes interactively target the Wnt signaling factors. While the BAF complex may exhibit selective (inhibition and activation) regulatory effect on Wnt gene targets through direct interaction with β-catenin (Barker et al., 2001; Ronan et al., 2013; Son and Crabtree, 2014; Vasileiou et al., 2015), the polycomb complex antagonizes Wnt signaling to suppress neurogenic fate acquisition during neural development (Hirabayashi et al., 2009). Implying, at least in part, that in the absence of BAF complex, generation of neurons may be fundamentally disturbed during development of the nervous system. The next few sections in this review detail how BAF complex singularly or concertedly drives various aspects of neural development.

### BAF complex is indispensable in neural tissue specification

Lethality associated with complete knockout models of BAF complexes has posed major challenges in assessing the biological significance of loss of BAF subunits during development. Recently it was found that conditional knockout mouse models of BAF complex subunits, BAF155 and BAF170, resulted in dissociation of other BAF complex subunits, and hence disassembly of the entire complex. The free subunits were then degraded by the ubiquitin-proteasome system resulting in loss of BAF complex functionality (Narayanan et al., 2015). By applying the above mouse model system, the activity of chromatin remodeling BAF complex has been shown to be vital for the overall formation of nervous system structures, because loss of their functions caused severe neural tissue agenesis (Narayanan et al., 2015; Bachmann et al., 2016; Nguyen et al., 2016).

Following loss of BAF complex due to FoxG1-Cre-mediated double conditional deletion of the scaffolding subunits, BAF155 and BAF170, in the telencephalon, the entire forebrain and related structures including the olfactory bulb failed to develop (Bachmann et al., 2016). Similarly, though Emx1-Cre-mediated ablation of BAF complex also had severe effects on specification of cortical structure, the cortex formed thereof was flimsy and markedly malformed (Narayanan et al., 2015).

Induction of FoxG1-Cre activity via tamoxifen injection 3 days after the formation of primary NSCs caused loss of Sox2^+^ cells in olfactory placode and epithelium of BAF155/BAF170_dcKO mutants that were immunohistologically examined at developmental stages E9.5, E10.5, and E11.5. Consistently, there was an absolute lack of HuCD^+^, Tuj^+^, and Ctip2^+^ neurons in the olfactory placode/epithelium, hence confirming that olfactory epithelium (OE) was virtually not specified. Given that Pax6-dependent transcriptional activity was greatly reduced in dcKO of BAF155 and BAF 170 in olfactory NSCs, it has been mechanistically postulated that the BAF complex is essential for conventional activation of Pax6-dependent transcriptional activity in neural precursor cells (Ninkovic et al., 2013; Tuoc et al., 2013a; Bachmann et al., 2016).

### NSC proliferation is tightly modulated by activities of BAF complex

The mammalian nervous system consists of billions of neurons and glia that are predominantly derived from multipotent NSCs. NSCs initially exit as neuroepithelial cells (NECs), which later in early neural development (~E9.5–E10.5) transform into the so called multi-/unipotent radial glia cells (RGCs) located in the germinal zone of the brain and spinal cord. During development of the brain and spinal cord, RGCs proliferate via symmetric mitotic cell division to amplify their stemness pool from which subsequent neuronal and glial cell type diversity originates (Guillemot, 2007; Kriegstein and Alvarez-Buylla, 2009; Taverna et al., 2014; Tuoc et al., 2014).

Epigenetic alterations, including chromatin remodeling at specific genomic locations, have been correlated with differential expression of transcription factors, leading to temporal regulation of specific genes that define cell autonomous paradigms needed to establish NSC fate (Hirabayashi and Gotoh, 2010; Juliandi et al., 2010; Coskun et al., 2012; Ronan et al., 2013; Narayanan and Tuoc, 2014; Watson and Tsai, 2016; Yao et al., 2016). These alterations are necessary to maintain NSCs in the cell cycle so as to generate the appropriate number of progeny that will later differentiate to afford the right number of neurons and glia needed for normal neural tissue development. The BAF complex has emerged as a key determinant in the selective activation of developmental signals that ensure NSCs proliferate within a developmental time window before switching to differentiation mode.

Several experiments have led to the concept of the existence of specific npBAF complex (Lessard et al., 2007; Bachmann et al., 2016). The npBAF complex is exclusively functional in neural stem/progenitor cells, and is composed of either a BRG1 or BRM core ATPase, a BAF155::BAF155 homodimer or BAF155::BAF170 heterodimer, and BAF250 paralogs (BAF250A or BAF250B). This is in contrast to esBAF complex that is made up of only BRG1 ATPase with functionally non-redundant BAF155 and BAF170 subunits. In both cases however, subunits such as BAF45a/d, BAF53a, and BAF55a remain unchanged, and during neural induction are capable of specifying self-renewal and proliferation (Lessard et al., 2007; Staahl et al., 2013; Bachmann et al., 2016). Thus, esBAF complex is plausibly reconstituted in NSCs to form npBAF complex in order to confer multipotency, while maintaining proliferative ability. However, as neural development advances, say neural progenitors differentiating into neurons, npBAF complex switches subunits to form neuron-specific nBAF complex (Lessard et al., 2007; Bachmann et al., 2016), which is discussed further in the next section.

In a recent study, it was found that olfactory neural stem cells (oNSCs) express a specific BAF complex (onscBAF) that is essential for their proliferation and maintenance during the development of the OE, a peripheral aspect of the olfactory system (Bachmann et al., 2016). Even though it was apparent (as per Ascl1 and Ngn1 progenitor-specific, and HuC/D, Tuj, and Lhx2 immature neuron-specific immunostainings) that neurogenesis at early (~E10.5) but not later (~E13.5) stages in FoxG1 BAF155 mutants was not significantly affected, there was striking reduction in Pax6^+^, Nestin^+^, and Sox2^+^ oNSCs in the OE basal layer. By applying various cell cycle markers, it was ascertained that progenitor proliferation and cell cycle exit index was greatly reduced in BAF155 mutants. Interestingly, proliferation of apically located Pax6^+^, Sox2^+^, Otx2^+^, REEP6^+^, and K18^+^ glial-like sustentacular cells were unaffected by loss of BAF155 in the olfactory placode or epithelium (Bachmann et al., 2016). Explanatorily, loss of BAF155 subunit during prenatal development of the mouse OE caused precocious regression of NSCs proliferative capacity, leading to reduced generation of neural precursors and hence neurons. Consequently, neurogenesis was impaired at later developmental stages and resulted in dysgenesis of the OE. The function of BAF155 in NSCs to a large extent seem to be evolutionally conserved as its homolog, psa-4, together with psa-1 (BRG1) in worms are known to be important for asymmetric division of NSCs (Bultman et al., 2000; Sawa et al., 2000; Kim et al., 2001).

Direct observation of neurosphere formation in culture indicated impaired proliferative and self-renewal capacity in NSCs lacking BRG1. To that end, *In vivo* Cre-directed deletion of BRG1 under Nestin promoter in mouse NSCs at E10.5 led to reduced brain size, consequent to diminished proliferation and depletion of neural precursor population. Perinatal lethality was thus inevitable (Matsumoto et al., 2006; Lessard et al., 2007). Compelling evidence obtained from knockdown investigations indicates that other BAF subunits, such as BAF45a/d, BAF53a, and BAF55a are also indispensable for NSC proliferation and self-regeneration (Lessard et al., 2007; Staahl et al., 2013).

Various signaling pathways known to drive cell proliferation are engaged during BAF complex-mediated transcription regulation of neural development (Zhan et al., 2011; Vasileiou et al., 2015). For instance, BRG1-containing npBAF interacts with components of the Wnt signaling pathway to promote neural progenitor proliferation in the telencephalon (Vasileiou et al., 2015). Also during neural patterning, npBAF complex activates aspects of the notch signaling cascade leading to proliferation of CNS precursor cells, while suppressing the sonic hedgehog signaling pathway (Zhan et al., 2011).

### Role of BAF complex in neurogenesis

During embryonic and early post-natal neural development, proliferative and multipotent NSCs acquire unipotent fate and ultimately differentiate to generate neurons in the process of neurogenesis. In the mammalian embryonic brain, two major neurogenic (germinal) zones, ventricular and subventricular zones (VZ/SVZ), are the main sites of neurogenesis. The VZ abuts the lateral ventricles whereas the SVZ is atop the VZ (Guillemot, 2007; Kriegstein and Alvarez-Buylla, 2009; Taverna et al., 2014; Tuoc et al., 2014). Some neurogenic centers persist in the adult brain to ensure what is known as adult neurogenesis (Zhao et al., 2008; Ming and Song, 2011).

Neuronal generation, identity establishment, and migration to final functional areal position are under multiple regulatory factors including chromatin remodelers. In this section, we will describe the crucial involvement of the chromatin remodeling BAF complex in orchestrating the transition of neural stem/progenitor cells to fully differentiated neurons, and their final localization during early and late neural development.

#### BAF complex drives embryonic neurogenesis

During CNS embryogenesis, such as cortical development, multipotent neural stem cell-like RGCs located in the VZ are able to undergo symmetric mitotic divisions to amplify their pool, or asymmetrically to generate lineage-restricted progeny (Noctor et al., 2004; Guillemot, 2007; Kriegstein and Alvarez-Buylla, 2009; Taverna et al., 2014; Tuoc et al., 2014). Early during embryonic neurogenesis, RGCs divide to directly produce neurons, and later indirectly to produce neurogenic precursors known as intermediate progenitors (IPs) (Haubensak et al., 2004; Miyata et al., 2004) in lissencephalics (lacking cortical folding) and gyrencephalics (with convoluted cortex), as well as outer radial glia (oRG) progenitors that reside in the SVZ and outer SVZ, respectively (Haubensak et al., 2004; Miyata et al., 2004; Hansen et al., 2010; Wang et al., 2011; Borrell and Gotz, 2014; Wong et al., 2015).

As these neuronal stem cell-like precursors acquire neuronal fate, they switch from proliferative to neurogenic cell division mode in the telencephalon. As a result, there is a corresponding shift in epigenetic paradigms including competitive subunit dynamics and BAF complex re-composition, leading to npBAF complex transition to nBAF complex (Lessard et al., 2007; Wu et al., 2007; Bachmann et al., 2016). So far, three key subunit substitutions have been observed in the npBAF-nBAF complex transition: BAF45a/d are swapped for BAF45b/c, BAF53a for BAF53b, and BAF55a for BAF55b; as well as change in expression level of BAF155 and BAF170 (Lessard et al., 2007; Wu et al., 2007; Bachmann et al., 2016).

Mechanistically, Yoo et al. (2009) have ascribed the BAF complex subunit reconstitution phenomenon during neural development (especially for BAF53a/b transition) to activities of two important microRNAs (miR9^*^ and miR124) that are selectively expressed in post-mitotic neurons. That is, this microRNA-mediated repression of BAF53a may be responsible for the activation of its variant BAF53b exclusively found in the nBAF complex. In that direction, they affirmed previous findings that miR9^*^ and miR124 are antagonistic targets of the repressor-element-1-silencing transcription factor [REST] (Conaco et al., 2006). This suggests a possible mechanism of BAF53a/b switching in which REST is recruited to derepress the direct suppressive effect of miR9^*^ and miR124 on BAF53a. While these findings extend our understanding of BAF complex dynamics, they are provocative enough to warrant further investigations aimed at dissecting the switching mechanisms of other BAF subunits which lead to npBAF-nBAF transition that critically coincides with cell cycle exit of multipotent neuronal precursors to become more differentiated.

The BAF complex has also been shown to be an influential factor in determining the mode of embryonic neurogenesis; that is whether by direct generation of neurons from RGCs (direct neurogenesis) or indirectly from IPs (Tuoc et al., 2013a,b). Indirect neurogenesis has been shown to result in expansion of neuronal progenitor pool, and hence contribute to evolutionary cortical expansion and complexity. During development of nervous tissues, BAF complex interacts with the transcription factor, Pax6 that principally regulates neurogenesis. Competitive dynamics of BAF155 and BAF170 subunit incorporation into npBAF complex regulates chromatin state and tendency of recruiting the transcription repressor, REST, to Pax6 gene. Notably, *In vitro* studies show that, while BAF170 subunit has REST binding motif in its C-terminus, BAF155 bears divergent amino acid sequence in its C-terminus, and hence unable to bind and recruit REST (Tuoc et al., 2013a). Early in neocorticogenesis (E12.5–E14.5), BAF170-incorporated BAF complex repressively interacts with Pax6 by creating a heterochromatin state, which restricts the expression of Pax6 target genes such as Tle1, Cux1, and Tbr2, and consequently promotes direct neurogenesis. However, beyond E14.5, BAF170 expression decreases while BAF155 expression is augmented. This promotes euchromatin state and activates transcription of the aforementioned Pax6 target genes; thereby leading to enhancement of indirect neurogenesis and hence increase in basal progenitor pool (Tuoc et al., 2013a,b). *In vivo* conditional loss of BAF170 in neocortical progenitors using an Emx1-Cre causes aberrant expansion in IP pool with attendant increased propensity toward upper cortical layer fate. These happen to be effects converse to Pax6 deletion. On the other hand, BAF170 gain of function has phenotypic effects including upper cortical layer neuron depletion similar to Pax6 null (Tuoc et al., 2013a).

#### Control of adult neurogenesis by BAF complex

After birth, neurogenesis persists in limited regions of the mammalian CNS, constituting the so-called adult neurogenesis (Zhao et al., 2008; Ming and Song, 2011). Adult neurogenic regions are referred to as stem cell niches because they contain NSCs in specialized microenvironment (Zhao et al., 2008; Ming and Song, 2011). There are two major NSC niches defined so far in adult mouse brain: subgranular zone (SGZ) of the dentate gyrus (DG) and the subependymal zone (SEZ) of the lateral ventricles, from where neuroblasts migrate rostrally to populate the olfactory bulb (OB) and also supplies neurons to striatal areas throughout life (Zhao et al., 2008; Ming and Song, 2011).

Few important studies have shown that components of the BAF complex promote adult neurogenesis. In the most recent study, BAF170 subunit was found to be expressed in radial glia-like (RGL) cells and other adult neurogenic cell type in the hippocampal formation. Conditional loss of BAF170 in adult murine brain led to depletion of RGL precursor pool while promoting astrocytic fate. The resultant imbalance in adult neurogenesis was correlated with cognitive dysfunction (Tuoc et al., 2016).

In another study, it was found that expression of the BAF subunit, Ctip2 (Bcl11b), by post-mitotic granule neurons was important for the post-natal development of the dentate gyrus and hippocampus. By using forebrain specific Nex- and Emx1-Cre mediated ablation of Ctip2, it was shown that loss of Ctip2 expression in the adult hippocampus and dentate gyrus caused reduction in neural progenitor pool, neuron generation, and impairment in neuronal differentiation. It was thus revealed that while Ctip2 expression by post-mitotic granule cells is required for their own differentiation, it exerts a paracrine effect by also regulating proliferation of progenitors in a feedback mechanistic fashion. A dual phase-specific Ctip2 functionality is hence proposed to be operational during adult neurogenesis in the hippocampal formation. Interestingly, re-expression of a direct transcriptional downstream effector of Ctip2, Desmoplakin (functions as cell adhesion molecule), is capable of reversing the said mutagenic phenotypes attributable to Ctip2 ablation in the adult hippocampus and dentate gyrus (Simon et al., 2012). Similarly, by using tetracycline-dependent induction of forebrain-specific Cre-mediated deletion of Ctip2 under the calcium/calmodulin-dependent protein kinase type II alpha chain (CaMKIIα) promoter, it has been corroborated that the post-natal expression of Ctip2 is crucial for the survival, differentiation, and circuit integration of granule cell neurons generated in the adult stages during hippocampal development (Simon et al., 2016). Altogether, the studies suggest that sustained post-natal expression of Ctip2 by differentiated granule cells is essential for the post-natal development of the hippocampus, which, as already mentioned, is a major center of cognition in the mammalian brain.

Evidence of the role of chromatin remodeling BAF complex in adult neurogenesis has also been deepened by findings that BRG1, but not BRM-containing BAF complex essentially interacts with Pax6 to specify neuronal identity of adult neural progenitors resident in the subependymal stem cell niche. The study showed that by directly interacting with Pax6, the BRG1-ATPase core of BAF complex is able to potentiate the recruitment of neurogenic transcription factors such as Sox11, Nfib, and Pou3f4 to Pax6 gene targets, thereby forming regulatory feedback network in orchestrating fate specification during adult neurogenesis. Adult NSCs lose neuronal fate tendency and become bias toward glial cell lineage upon deletion of BRG1 just as in the case of Pax6 ablation (Ninkovic et al., 2013; Petrik et al., 2015).

#### Genesis of neuronal subtypes is regulated by SWI/SNF (BAF) complex

A vast number of neurons with phenomenal diversity are formed during development of the nervous system. These neurons receive specific instructions in order to acquire unique neuronal identity. The cerebral cortex for example is made up of millions of projection/pyramidal neurons (PNs) broadly organized into several functional domains (Custo Greig et al., 2013; Harb et al., 2016). When PNs are newly generated from their precursors in the VZ/SVZ, they are differentially naive as they share widely similar and overlapping characteristics. However, through selective transcriptional activation and repression of a number of fate specifying genes and fine regulatory mechanisms, specific PN identities are acquired, yielding the phenomenal diversity in PN population in the post-natal cortex (Custo Greig et al., 2013).

Several studies elaborate on the pivotal involvement of the BAF complex in the specification and generation of neuronal subtype identity during neural development, and they also give accounts of various aberrant phenotypes attributable to the ablation of specific BAF complex subunits. Using an *in vivo* mouse model, it has been shown that the BRM-ATPase based BAF complex containing BAF155 and BAF170 subunits is able to bind to Pax6 in a time-regulated manner to modulate the expression of related gene targets such as Cux1, Tle1, and Tbr2. These genes are required for the generation and specification of upper layer neuronal identity (Tuoc et al., 2013a,b).

Fascinatingly, two BAF complex paralogous subunits, BAF100a (Ctip1/Bcl11a) and BAF100b (Ctip2/Bcl11b), have also been identified as crucial molecular determinants of PN subtype specification. While Ctip1 is strongly expressed by post-mitotic callosal and corticothalamic PNs, Ctip2 is solely expressed in subcerebral PNs and post-mitotic striatal medium spiny neurons during embryonic stages of cortical development. As such, Ctip1 has been reported to modulate precision of neocorticogenesis via regulation of factors that define subclass identity of deep-layer PNs, whereas Ctip2 expression is critical for corticospinal PN terminal differentiation and circuitry (Arlotta et al., 2005, 2008; Woodworth et al., 2016). Ablation of Ctip1 leads to preponderance of subcerebral PN development in sensory cortical areas over genesis of deep-layer callosal and corticothalamic PNs, hence indicating Ctip1 importance in establishing sensory areas in the cortex. *In vivo* Ctip1 gain-of-function (GOF) however, suppresses generation and axonogenesis of subcerebral PNs (Woodworth et al., 2016).

Various findings point to the fact that Ctip2 expression is controlled by other transcription factors in directing PN specification and terminal differentiation. For instance, the transcription factor Fezf2 promotes the expression of Ctip2 to synergistically control differentiation of neurons that project to subcerebral targets, while adequately restricting the expression of other neuronal subtype identity-specifying transcription factors (e.g., Sox5, Satb2, and Trb1) (Arlotta et al., 2005; Molyneaux et al., 2005; Chen et al., 2008; Bedogni et al., 2010). It implies that co-repression of Ctip2 and Fezf2 is necessary to permit other neuronal subtype generation. For example, repression by Sox5 is critical for establishing neocortical layer 5/6 (lower layer) PN identity (Kwan et al., 2008; Lai et al., 2008; Shim et al., 2012), whereas repression by Satb2 yields upper layer PN identity (Alcamo et al., 2008). Suppression of Ctip2 by Tbr1 is also known to determine layer 6 (corticothalamic) PN subclass (Bedogni et al., 2010; Han et al., 2011). Neuronal subtype identity is however a continuous process that may involve further modulatory/tweaking factors even at late embryonic stages (Azim et al., 2009).

Loss of Ctip2 in the brain interferes with normal differentiation of subcerebral PN. Mainly, axonal growth, trimming, and pathfinding is affected in the absence of Ctip2 expression (Arlotta et al., 2005). In the hippocampus, null mutation of Ctip2 causes dispersion of neurons that under normal conditions are kept in the confinement of the granular layer. These neurons also display impaired dendritogenesis and decreased expression of calbindin (Wu et al., 2007).

### BAF complex orchestrates neuronal migration, maturation, and dendritic morphogenesis

Accompanying the generation of neurons from progenitors during neurogenesis, immature neurons usually undergo different forms of migration (e.g., radial, tangential, and multipolar) from their birthplaces to their final functional location in the developing brain (Evsyukova et al., 2013). In the developing neocortex, newly born neurons predominantly migrate radially under glial guidance out of the VZ or SVZ to form the laminated cortical plate.

Various factors regulate neuronal migration in the developing cortex (Evsyukova et al., 2013). Although much is yet to be known about the role of chromatin remodeling factors in neuronal migration, Wiegreffe et al. (2015) have elegantly provided striking evidence showing that the chromatin remodeling BAF complex subunit, BAF100a (Ctip1/Bcl11a), is crucial in regulating radial migration of PNs in the developing cortex. Importantly they indicated that Ctip1 expression by migrating neurons (Leid et al., 2004) in the intermediate zone (IZ) is required for multipolar to bipolar morphology transition, which is a critical stage in the process of radial migration (Wiegreffe et al., 2015). After electroporating Cre-GFP into BAF100a-flox/flox brains at E14.5, more GFP-positive multipolar neurons compared to bipolar neurons were found in the IZ of E17.5 neocortex. The multipolar cell accumulation in the IZ may signify failed or disrupted morphological switch to continue their radial migration to cortical layers. Most of the aberrantly migrating neurons due to Bcl11a deletion displayed disorientation with respect to the pial surface, which is an opposing phenotype to normal radially migrating neurons (Wiegreffe et al., 2015). At post-natal stages, BAF100a-deficient cortex displayed discernable dysplasia, especially in the upper layers with resultant reduction in inter-hemispheric connection. Mechanistically, it was revealed that BAF100a directly regulated its downstream effector Sema3c to control PN polarity and radial migration that ensured proper formation of upper cortical layers during neocorticogenesis (Wiegreffe et al., 2015).

After gaining subtype identity, immature neurons undergo specific differentiation processes to become fully matured and functional. During neural maturation, neurites are specified to become either axons or dendrites with which neurons make synaptic connections with adjoining neurons to afford circuit integration. Improper axonogenesis (axonal growth and pathfinding) and dendritogenesis (dendritic outgrowth, arborization, and refinement) results in anomalous neuronal morphology and synaptic connections that underscore several neurodevelopmental disorders. Many factors, including chromatin regulators play vital roles during neural morphogenesis (axonogenesis and dendritogenesis) (Whitford et al., 2002; Wu et al., 2007; Bachmann et al., 2016).

The BAF complex is important in neuronal morphogenesis during neural development (Whitford et al., 2002; Wu et al., 2007; John et al., 2012; Weinberg et al., 2013; Choi et al., 2015; Bachmann et al., 2016). BAF complex controls specification of neurites to differentiate into axons, usually one per neuron, and subsequent axonal fasciculation and pathfinding (Weinberg et al., 2013; Bachmann et al., 2016). For example, sensory neurons found in the dorsal root ganglion (DRG) extend axons that synapse with second order neurons in the dorsal spinal cord to allow relay of somatosensory information. It has been revealed that the BAF complex subunit, BAF100a, is required for terminal maturation and morphogenesis of dorsal spinal neurons during development of the spinal cord (John et al., 2012). Loss of BAF100a expression in spinal neurons was shown to cause central axon dysgenesis, leading to denervation of the dorsal horn by primary somatosensory neurons in the DRG.

Post-mitotic neuron specific BAF53b subunit (Olave et al., 2002) has been shown to play a key role in activity-dependent dendritic morphogenesis. Homozygous knockout of BAF53b subunit in mice largely led to early post-natal lethality, with just about 10% surviving up to adult stages, albeit they displayed behavioral disturbances such as hyperactivity (Lessard et al., 2007; Wu et al., 2007). Cultured cortical neurons lacking BAF53b (BAF53b^−/−^) exhibited striking activity-dependent dendritogenic incompetence, which phenotype could only be rescued by exogenous BAF53b GOF but not its homolog, BAF53a (Wu et al., 2007). To mutually exclude the functional role of BAF53b from its homolog, BAF53a, in orchestrating dendritogenesis, two chimeric protein candidates, BAF53a with subdomain 2 of BAF53b and its reverse were generated. Intriguingly, the chimeric BAF53a protein containing subdomain 2 of BAF53b, but not its reverse candidate was able to affect activity-dependent dendrite outgrowth, hence suggesting the divergent subdomain 2 of BAF53b as indispensable for activity-dependent dendrite formation (Wu et al., 2007). That notwithstanding, phenotypes similar to BAF53b^−/−^ were observed when other subunits of the nBAF complex (i.e., BRG1, BAF45b, and BAF57) were knocked down using RNA interference (Lessard et al., 2007; Wu et al., 2007), suggesting multiple BAF subunit involvement in neurite development. The outcome of investigations in invertebrate models recapitulates BAF53b as a critical player in neuronal dendritogenesis. Loss of function of BAP55 (homolog of BAF53a/b) in *Drosophila melanogaster* interfered with normal dendritic targeting of olfactory projection neurons, which phenotype was rescuable with expression of mammalian BAF53a/b using GH146-Gal4 driver (Tea and Luo, 2011). Additionally, loss of BAP55 and other *Drosophila* homologs of BRM, BAF60c and BAF47, perturbed formation of sensory neural dendrites in the peripheral nervous system (Parrish et al., 2006). Plausible mechanisms underlying the role of BAF complex in orchestrating dendritic elaboration include the loss of BAF53b, which causes reduction in the entire BAF complex occupancy at promoters of dendritogenic genes such as GAP43 and Ephexin1, hence culminating in alterations in their expression (Wu et al., 2007). BAF complex is also known to mechanistically engage the calcium-mediated transcription activation function of calcium-responsive transactivator (CREST), which links activity responsive Ca^2+^ signaling and chromatin reorganization by BAF complex known to lead to dendritic outgrowth, arborization, and refinement (Aizawa et al., 2004; Wu et al., 2007).

Even though early olfactory receptor neuron (ORN) population identified by Lhx2^+^, Tuj^+^, and HuCD^+^ immunostaining did not change significantly in BAF155 knockout mice, axonogenesis was markedly defective at early embryonic stage (E13.5) (Bachmann et al., 2016). Likewise, the high expression of BAF170 in ORNs is essential for their maturation, because diminished number of OMP^+^ mature ORN was found in BAF170-deficit OEs (Bachmann et al., 2016). Traditionally, immature ORNs located in the developing OE project axons that synapse with the dendrites of mitral cells in the olfactory bulb during differentiation. The afferent pathway so formed then permits second order axonal projection from mitral cells to primary olfactory centers in the cortex. To confirm interrupted axonal pathfinding from ORNs to telencephalic regions due to the absence of BAF155 subunit, Dil crystal injection of OE was used to reveal axonal dysgenesis in BAF155 deficit ORNs as opposed to obvious visualization of Dil-labeled olfactory neuronal tract in controls (Bachmann et al., 2016). The observation thus consolidates the idea that BAF155 subunit is important for neuronal axonogenesis in the olfactory system. The significance of BAF complex in directing axonal pathfinding is also observed in *Caenorhabditis elegans* as deletion of the nematode homolog of BAF60 (ham-3) disturbed axonal projection during differentiation of some serotonin-producing neurons (Weinberg et al., 2013).

Thus, the BAF complex plays crucial roles in neuronal morphogenesis in different parts of the CNS (e.g., dorsal root ganglion, forebrain, and olfactory system) in both invertebrates and vertebrates.

### BRG1-containing BAF complex regulates gliogenesis

Gliogenesis is a neurodevelopmental process that leads to the generation of non-neuronal glial cell types including astrocytes and oligodendrocytes from multipotent NSCs. The production of astrocytes (astrogenesis) and oligodendrocytes (oligodendrogenesis) have been extensively studied in the developing brain and spinal cord (Rowitch and Kriegstein, 2010; Gallo and Deneen, 2014). In many structures of the CNS such as the cortex, differentiation of NSCs initially primed to generate neurons at early stages become progressively restricted in the course of cortical development. Typically, NSCs in the cortex undergo intrinsic changes that make them acquire glial fate at later stages of neural development, with astrogenesis peaking at P0–P2 and oligodendrogenesis at late post-natal stages around P14 in mice (Freeman, 2010; Rowitch and Kriegstein, 2010; Gallo and Deneen, 2014). Epigenetic cell-type-specific regulation of gene expression is implied in changes in NSCs that determine neuro/gliogenic fate. These include transcription factor expression selectivity engendered by epigenetic alterations such as chromatin remodeling that control NSC fate (Hirabayashi and Gotoh, 2010; Juliandi et al., 2010; Coskun et al., 2012; Narayanan and Tuoc, 2014; Yao et al., 2016).

Although the interest in astrocyte function has increased dramatically in recent years, our understanding of astrocyte development, especially under the control of epigenetic factors, has lagged behind as compared to other brain cell types. In addition, the molecular mechanisms underlying astrocyte specification and expansion are largely unknown. Phenotypic analyses of early loss of BRG1 in BRG1cKO mutants with Nestin-Cre indicated that the loss of this core BAF subunit led to a severe defect in the maintenance of NSC pool and consequently also in the production of neurons and glia cells (Matsumoto et al., 2006; Lessard et al., 2007). However, it is unknown whether chromatin remodeling via the BAF-complex regulatory function might control embryonic astrogenesis. In post-natal brain, BAF complex is apparently required in permitting neurogenic tendencies of NSCs, while suppressing their gliogenic commitments. Hence, when BRG1 (Ninkovic et al., 2013) or BAF170 (Tuoc et al., 2016) were abolished in adult NSCs, there was premature onset of gliogenesis.

Oligodendrocyte identity specification is critical to establishing myelination schemes during CNS development. The chromatin remodeling BAF complex also finely regulates generation and maturation of oligodendrocytes. The BRG1 ATPase core of the BAF complex is reported to be vital for the generation of oligodendrocyte lineage. Multi-stage chromatin immunoprecipitation (ChIP) sequencing studies reveal that co-occupancy of BRG1 and the oligodendrocyte-lineage specifying basic-helix-loop-helix transcription factor, Olig2, at enhancer elements synergistically regulate recruitment of transcription factors to gene loci central to oligodendrogenesis (Yu et al., 2013; Bischof et al., 2015). Matsumoto et al. (2006) went further to elucidate how BRG1 interacts with Olig2 to specify oligodendrocytic fate in neural progenitors at early cortical developmental stages. They reported that at about E14 in the mouse cortex, at which stage oligodendrocyte precursors are yet to colonize the cortex, BRG1 was found to interact with Olig2 promoter, leading to Olig2 transcriptional repression. As such, nestin-dependent deletion of BRG1 in early neural progenitor cells resulted in premature expression of Olig2 in the cortex, although such precocious Olig2 expressing cells could not differentiate into oligodendrocyte precursor cells (OPCs). That notwithstanding, BRG1 was found to interact with the said proximal Olig2 promoter in the ganglionic eminence where neural progenitors normally transform into Olig2^+^ OPCs, implying that the BRG1 component of the BAF complex is a repressor of Olig2 expression and an indirect suppressor of OPC specification in the early developing cortex. However, it is needed for the differentiation and maturation of OPCs (Matsumoto et al., 2006; Yu et al., 2013; Bischof et al., 2015).

### BAF complex function in cognition: learning and memory

Learning and memory (formation and storage) are cognitive functions that are underscored by persistent alterations in memory-related neural circuits due to transient environmental stimuli that result in behavioral changes (Day and Sweatt, 2010; Fischer, 2014; Watson and Tsai, 2016). The hippocampal formation is involved in cognitive functions such as memory formation, fear conditioning, and learning (Goncalves et al., 2016). The evidence thereof is mostly centered on hippocampal and DG neurogenesis (adult neurogenesis), dendritogenesis of hippocampal neurons, and various hippocampal-dependent activity experimentations in which synaptogenesis and synaptic alterations such as long-term potentiation (LTP) have been used to study memory formation and maintenance (Goncalves et al., 2016). Overall generation and integration of new neurons into complex neural circuits at pre-/post-natal developmental stages are also of great importance in memory formation and behavior (Deng et al., 2010; Urban and Guillemot, 2014). Given that the hippocampus is a major center of cognitive function in the brain, neurogenic activities that lead to its formation is of critical value to learning and memory. Hippocampal structures, such as the hippocampus proper and the DG are malformed in the presence of defective neurogenesis, which are strongly correlated with behavioral imbalances.

Various regulatory factors play importance roles in learning and memory. For instance, long-term memory processing requires fine interplay of intracellular signaling with transcriptional, translational, and epigenetic factors to regulate gene expression in cognitive function development (Deng et al., 2010; Urban and Guillemot, 2014; Goncalves et al., 2016). Epigenetic mechanisms such as methylation, acetylation, phosphorylation, ribosylation, and chromatin remodeling state (heterochromatin or euchromatin) regulate memory formation and maintenance (Day and Sweatt, 2010; Fischer, 2014; Watson and Tsai, 2016). Accumulating evidence from recent studies indicates the essentiality of the chromatin remodeling BAF complexes in memory formation and learning. Dysfunction of subunits in both npBAF and nBAF complexes in the CNS has been posited to underline various psychiatric perturbations (Koga et al., 2009; Neale et al., 2012; Yoo et al., 2017).

As already stated, BAF170 subunit in BRG1-containing BAF complex is expressed in RGL progenitors in the DG of the adult brain. For this reason, when hGFAP-Cre and Nestin-CreER-mediated conditional loss of BAF170 in RGL cells was performed, it caused abnormal proliferation and localization of RGL cells, with subsequent depletion of progenitor pools in the SGZ of the DG. Due to the lack of BAF170 in RGL progenitors, neurogenesis in DG was skewed to terminal astrogenesis. By using the Morris water maze test, BAF170 mutant mice were observed to display noticeable impairment in adaptive behavior (Tuoc et al., 2016).

The nBAF subunit, BAF100b (Ctip2/Bcl11b), is exclusively expressed in post-mitotic granule neurons in the SGZ of the DG but not in progenitors. Its expression is important for embryonic and adult hippocampal neurogenesis leading to proper functioning of the hippocampus in memory and learning. Newly born neurons lacking Ctip2 locate ectopically of the granule cell layer in the hippocampus. These Ctip2-deficient neurons characteristically display reduced expression of calbindin 1 (Calb1), which plays important roles in neuro-behavioral and memory formation (Simon et al., 2012; Soontornniyomkij et al., 2012). On the whole, loss of Ctip2 during development of the hippocampus leads to dramatic reduction in size and neuronal pool of the DG, and impairment of spatial memory formation (Simon et al., 2012, 2016; Lennon et al., 2017).

Another nBAF subunit, BAF53b (Vogel-Ciernia et al., 2013; Choi et al., 2015; Yoo et al., 2017) is also shown to play a strong role in cognitive processes in mice. Upon RNA sequencing of the dorsal hippocampus of BAF53b^+/−^ mutant mice, it was revealed that genes involved in memory formation (including cytoskeletal reorganizers and chromatin modifiers) were misregulated when compared with wild-type mice (Vogel-Ciernia et al., 2013). Consistently, alterations in the neuron-specific BAF complex subunit BAF53b expression in amygdala neurons modulated learning and memory formation in adult mice. Specifically, when Yoo et al. (2017) applied viral vector-mediated methods to knockdown or overexpress BAF53b functionality within the lateral amygdala in experimental auditory fear conditioning schemes, they found that inductive expression of BAF53b accompanied learning activities to augment memory consolidation and related neuroplasticity. Likewise, disrupted normal levels of BAF53b expression by genetic manipulations leading to BAF53b heterozygosity (BAF53b^+/−^) in mouse CaMKIIa^+^ post-mitotic neurons, caused aberrant neuronal morphology acquisition such as defective spine formation, severe defects in hippocampal synaptic plasticity, and long-term memory impairment (Vogel-Ciernia et al., 2013).

Similar to phenotypes of BAF53b mutant mice, mutation in CREST (also known as SS18L1, a newly identified nBAF subunit) causes defects in learning and memory. Mechanistically, CREST acts together with the co-activator, CREB-binding protein (CBP), to regulate expression of immediate early genes including c-fos. Such early genes play significant roles in brain plasticity, activity dependent memory formation, and other complex behaviors.

All together, these findings suggest that BAF complex-driven chromatin remodeling in neuron generation, differentiation, synaptic development, and plasticity in related structures such as the hippocampus is essential for cognitive functions.

## Role of BAF complex in neurodevelopmental disorders

Given that the BAF complex plays crucial roles in neural development, it is clear to rationalize how its misregulation culminates in both syndromic and non-syndromic neurological problems. As such, exome sequencing analyses have implicated *de novo* BAF complex mutations as the underlying cause of some cognitive and psychiatric disturbances reported in humans (Figure 1, Table 2). Notably, while some polymorphic forms of BAF complex subunits cause more general and/or overlapping defective neurological phenotypes, others have been strongly linked to mutually exclusive intellectual disabilities and related non-neurological problems. Common and most characterized of these BAF complex-related neural disorders include the following discussed syndromes and conditions.

**Table 2:** Mutated components of BAF complex in human mental disorders.

**Gene name**	**BAF subunit**	**Mutation**	**Human mental disorders**	**Core phenotypes**	**References**
ARID1A	BAF250a	Nonsense, frameshift indel	Coffin–Siris syndrome	Coarsening of facial features, hypoplasia of the fifth finger/toe nails	Tsurusaki et al., 2012
ARID1B	BAF250b	Translocation, frameshift indel, nonsense, missense, microdeletion	Intellectual disability, Coffin–Siris syndrome, autism, Nicolaides-Baraitser syndrome, schizophrenia, Hirschsprung's disease	Coarse facial features, hypoplastic-to-absent nail of the fifth finger or toe; prominence of the interphalangeal joints and distal phalanges; social deficits and communication difficulties; delusion, thought disorder, auditory hallucination	Backx et al., 2011; Nord et al., 2011; Halgren et al., 2012; O'Roak et al., 2012; Santen et al., 2012; Smith et al., 2016; Takenouchi et al., 2016
ARID2	BAF200	Frameshift indel	Coffin-Siris syndrome	Coarsening of facial features, hypoplasia of the fifth toe nails	Bramswig et al., 2017
SMARCA2	BRM	Partial deletion, missense, intronic alteration, duplication	Coffin-Siris syndrome, Nicolaides-Baraitser syndrome, schizophrenia	Coarsening of facial features, hypoplasia of the fifth finger/toe nails; prominence of the interphalangeal joints and distal phalanges; delusion, thought disorder, auditory hallucination	Morin et al., 2003; Koga et al., 2009; Loe-Mie et al., 2010; Wolff et al., 2012; Ejaz et al., 2016; Tang et al., 2017
SMARCA4	BRG1	Partial deletion, missense	Coffin-Siris syndrome, autism	Coarsening of facial features, hypoplasia of the fifth finger/toe nails; social deficits and communication difficulties, restricted and repetitive behaviors	Tsurusaki et al., 2012; De Rubeis et al., 2014
SMARCB1	BAF47	In-frame deletion, missense	Coffin-Siris syndrome, Kleefstra syndrome phenotypic spectrum	Coarsening of facial features, hypoplasia of the fifth finger/toe nails; childhood hypotonia, and behavioral anomalies, synophrys, and midface hypoplasia	Kleefstra et al., 2012; Tsurusaki et al., 2012; Gossai et al., 2015
SMARCE1	BAF57	Missense	Coffin-Siris syndrome	Coarsening of facial features, hypoplasia of the fifth finger/toe nails	Wieczorek et al., 2013; Kosho et al., 2014b; Zarate et al., 2016
SMARCC1	BAF155	Missense	Autism	Social deficits and communication difficulties, restricted and repetitive behaviors	Neale et al., 2012
SMARCC2	BAF170	Splice site mutation	Autism	Social deficits and communication difficulties, restricted and repetitive behaviors	Neale et al., 2012
PBRM	BAF180	Missense	Autism	Social deficits and communication difficulties, restricted and repetitive behaviors	O'Roak et al., 2012
BCL11A	BAF100a BCL11A	Micro deletion, missense, and frameshift mutations	2p15–16.1 microdeletion syndrome; autism; schizophrenia	Social deficits and communication difficulties, restricted and repetitive behaviors; delusion, thought disorder, auditory hallucination	De Rubeis et al., 2014; Bagheri et al., 2016

### Coffin-siris syndrome (CSS)

Drs. Grange S. Coffin and Evelyn Siris were the first to report CSS (OMIM #135900) in 1970 (Kosho et al., 2014a). It is a rarely occurring congenital abnormality with clinical characteristics such as intellectual disability (ID), progressive coarsening of the face, hypertrichosis, frequent infections, feeding difficulties, and hypoplasia of the 5th distal phalanges and fingernails (Levy and Baraitser, 1991; Schrier et al., 2012). Schrier et al. (2012) summarized 80 previously reported cases for both common and discriminating clinical hallmarks, and brought forward a differential diagnosis between CSS and other related syndromes, including Nicolaides-Baraitser syndrome (NCBRS) and Deafness, Onychodystrophy, Osteodystrophy, Mental Retardation, and Seizures (DOORS) syndrome. In recent years, comprehensive human exome sequencing and genome-wide association studies have revealed that among roughly 180 CSS cases reported, about 110 patients carry a mutation in genes encoding BAF complex subunits. These genes include *BAF250A (ARID1A), BAF250B (ARID1B), BRM (SMARCA2), BRG1 (SMARCA4), BAF47 (SMARCB1), BAF57 (SMARCE1)*, and *BAF200 (ARID2)* (Santen et al., 2012; Tsurusaki et al., 2012; Bramswig et al., 2015, 2017; Miyake et al., 2016).

#### BAF250a/BAF250b

Up to date, eight CSS patients have been reported to harbor heterozygous mutations in *BAF250a* (Tsurusaki et al., 2012; Kosho et al., 2013, 2014a). Such mutations result in truncated BAF250a subunit protein, and are proposed to exert a loss-of-function mechanism. BAF250b, a homolog of BAF250a subunit, exists mutually exclusive to BAF250a in BAF complexes. It has been found that more than 70 patients with CSS (Kosho et al., 2014a; Mari et al., 2015), and >30 patients presenting non-syndromic ID carry BAF250b abnormalities (Kosho et al., 2014a), including cytogenetic abnormalities and point mutations (Table 2). BAF250b abnormalities account for 68–86% of CSS cases (Santen et al., 2013; Wieczorek et al., 2013; Tsurusaki et al., 2014). All of the reported BAF250b mutations involved in CSS resulted in related protein truncation, and are proposed to exert haploinsufficiency. CSS Patients from BAF250b abnormalities present variable developmental delays or ID, together with impaired speech. While BAF250a mutation is known to result in severer CSS clinical features with associated rigorous physical complications, BAF250b mutation-dependent CSS precludes typical characteristics such as coarsening of facial features, and with rather milder digital hypoplasia. The detailed comparison of clinical features of CSS patients from BAF complex subunit mutations has been reported (Kosho et al., 2014a).

**Figure 2:**
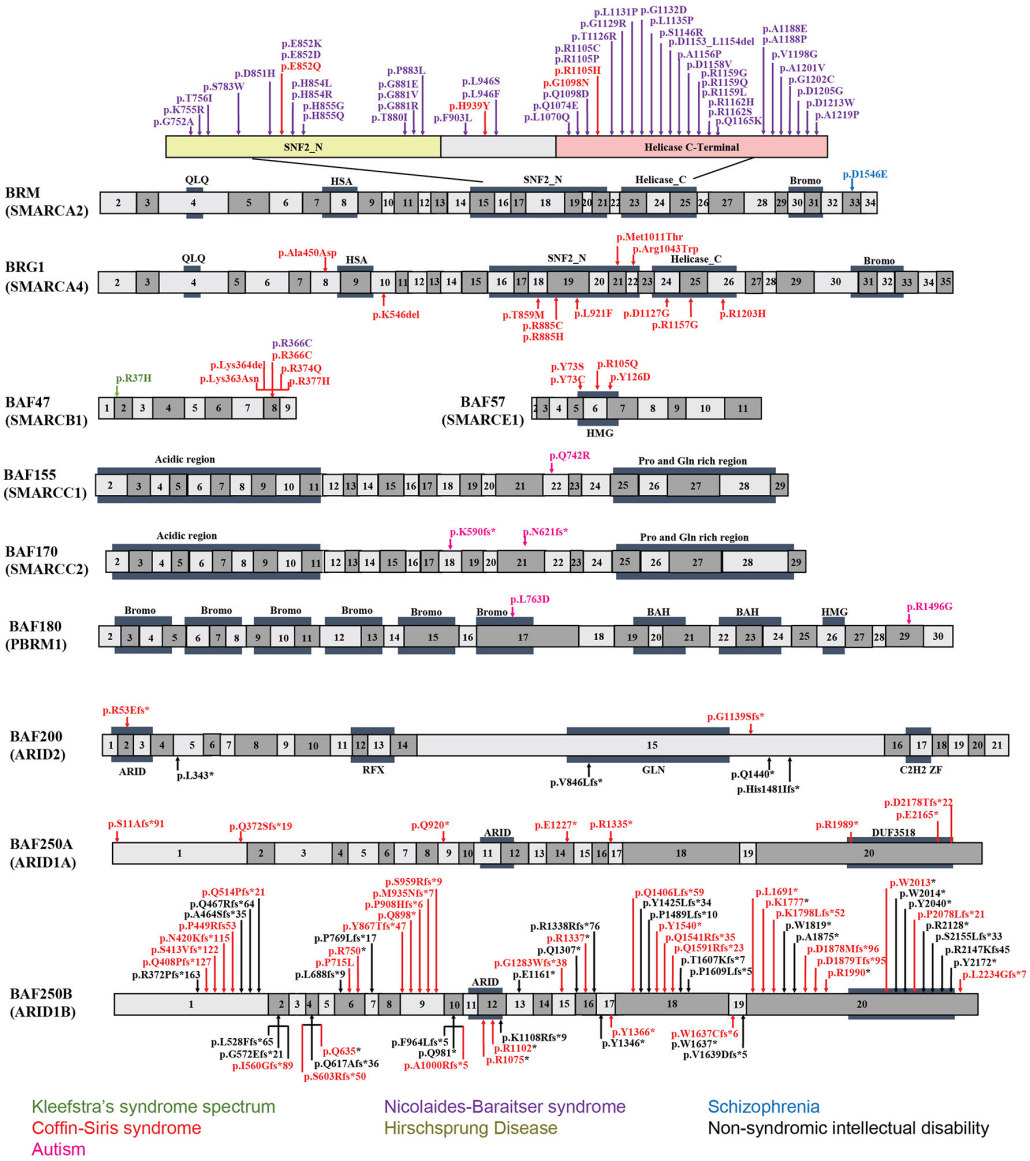
Exon-structure of the genes encoding for BAF subunits and sites of the pathogenic mutations in neurodevelopmental disorders. The specific functional domains are also shown: ARID, A-T rich interaction domain; QLQ, Gln, Leu, Gln motif; HSA, small helicase/SANT-associated domain; SNF, sucrose/non-fermenting domain; BROMO, bromodomain; HMG, high-mobility group domain; RFX, RFX-like DNA binding domain; C2H2 ZF, C2H2 zinc fingers.

BAF250a and BAF250b contain two characterized functional protein domains, an ARID in the center, and a domain of unknown function 3518 (DUF3518) at the C-terminus (Figure 2). ARID binds to AT-rich DNA with no obvious sequence specificity (Wang et al., 2004; Wilsker et al., 2004) and DUF3518 is required for interactions with BRG1 and BRM subunits in BAF complexes. Therefore, it is most likely that missense mutations in ARID will impair the ability of BAF250a/BAF250b to bind to chromatin DNA resulting in compromised BAF complex function, and that the missense mutations in DUF3518 may compromise the association of BAF250a (ARID1A) and BAF250b (ARID1B) with BRG1/BRM to form functional BAF complex (Sim et al., 2015). In line with this notion, it has been reported that the interactions of BAF250a with DNA are essential for SWI/SNF occupancy at promoters, and interruptions of which leads to embryonic lethality in mice (Chandler et al., 2013). Together, missense mutations from either domain would most probably exert a negative impact on BAF complex function. However, the mechanism behind the huge phenotypic variations is still not known. Interestingly, it has been proposed that the majority of nonsense and frameshift mutations result in mRNA degradation through nonsense-mediated mRNA decay (NMD) mechanism (Sim et al., 2015). Therefore, truncating mutations that escape from NMD in general leads to divergent and more serious phenotype than that found in NMD. However, further investigations are needed to clarify whether specific frameshift mutations in BAF250a/BAF250b result in NMD or truncated protein.

#### BRM/BRG1

*BRM* mutations, including four missense (Santen et al., 2013) and two duplication mutations (Miyake et al., 2016) have been observed in seven patients with CSS. As shown in Figure 2, the missense mutations were localized in the SNF2-ATPase domain. Therefore, these mutations of *BRM* most likely result in impairing the proper functioning of BAF complex by influencing ATP-hydrolysis. Remarkably, phenotypes from *BRM* mutations resulted-CSS highly overlap with that from NCBRS (Wieczorek et al., 2013), which sometimes make it difficult to make a clear diagnosis. In addition, it has been proposed that CSS could also derive from over-dosage of BRM, because two patients with a duplicated region including *BRM* presented typical CSS phenotype (Miyake et al., 2016).

Heterozygous mutations in *BRG1* have been observed in 12 CSS reported cases (Kosho et al., 2014a). As presented in Figure 2, the missense and in-frame deletions within SNF2-ATPase domain were also found in *BRG1* mutants in CSS patients. Most likely, the resulted non-truncating BRG1 proteins from the mutations were responsible for the phenotype, through GOF or dominant-negative effects (Kosho et al., 2014a). Although these two subunits have 74% similarity in amino acids sequence, *BRG1* mutations were exclusively linked with CSS, while *BRM* mutations were seen in both CSS and NCBRS (Wieczorek et al., 2013; Kosho et al., 2014a; Miyake et al., 2016).

#### BAF47/BAF57

To date, five pathogenic mutations have been found in *BAF47* in 13 CSS patients (Santen et al., 2013; Wieczorek et al., 2013; Tsurusaki et al., 2014). Interestingly, the mutations were all localized at three amino acid residues at positions 364, 374, and 377 within exons 8 and 9 (Figure 2), and the p.Lys364del mutation was frequently identified in nine patients, indicating the importance of these residues in normal BAF complex functionality. These non-missense or in-frame deletions result in truncated BAF47 protein products that lead to CSS phenotype through either dominant-negative or GOF mechanism. Recently, the emerging studies on genotype-phenotype correlation have found that CSS patients with *BAF47* mutations display the most striking physical features and rigorous problems associated with developmental delay, but with least distal limb anomalies (Mari et al., 2015).

Six CSS cases have been associated with *BAF57* pathogenic mutations, including pTyr73 Cys, pTyr73Ser, pArg105Gln, and pTyr126Asp (Tsurusaki et al., 2012; Santen et al., 2013; Wieczorek et al., 2013; Zarate et al., 2016). All of these cases have been reported to present with developmental delay in addition to moderate to severe ID. Moreover, they all have the typical features of CSS (Zarate et al., 2016). Notably, all these mutations are localized in the high-mobility-group (HMG) DNA binding domain (Figure 2). Although all mutations are located in the same domain, the resulted phenotypes display vast variation. For instance, abnormal brain structure was seen in only three out of six mutated cases. Therefore, how the missense mutations influence BAF complex function need further investigation.

#### BAF200

Mutation of BAF200 subunits was recently found to be associated with CSS. Recently, four patients with *BAF200* mutations were reported with non-syndromic ID, delayed development, and other clinical features (Shang et al., 2015). In another case, a patient with a duplication of *BAF200*, spanning exon 1–3, phenotypically displayed impaired language development and short stature (Zahir et al., 2016). More recently, two individuals bearing *de novo BAF200* frameshift mutations with typical CSS-like phenotypes including ID, coarse faces, and fifth toe nail hypoplasia were found (Bramswig et al., 2017). Based on the similarity of clinical phenotypes from the above patients, it has been proposed that *BAF200* mutations result in CSS as well (Bramswig et al., 2017).

### 6q25 microdeletion syndrome

The 6q25 microdeletion syndrome is an uncommon congenital and genetic disease characterized by ID with developmental delay, microcephaly, hearing impairment, distinct dysmorphic features, visual impairment, and corpus callosum agenesis (Santen et al., 2014; Ronzoni et al., 2016). Mutations of *BAF250b* were reported to be linked with this syndrome (Pirola et al., 1998). The case presenting with agenesis of corpus callosum (ACC) had a deletion of 5.5–7.6 Mb, including *BAF250b* and 10 other genes. To date, more than 24 cases with clear deletion information have been reported. Most of the patients carry deletions in *BAF250b* and several other genes, with deletion sizes of about 58 kb–14.5 Mb. Phenotypes are very variable, and dependent on the size and site of the deletion (Sim et al., 2015). It has been proposed that the haploinsufficiency of the affected genes results in defects in normal brain development and is responsible for the phenotype. The essential region for the phenotype was mapped to a 6q25.3 region harboring the protein-coding genes *ZDHHC14* and *BAF250b* (Michelson et al., 2012). Other studies (Halgren et al., 2012; Santen et al., 2014) further suggested that *BAF250b* was the critical gene responsible for the core phenotypes of the syndrome. Recently, Ronzoni et al., reported an evidence that loss of *BAF250b* mainly underpins 6q25 microdeletion syndrome (Ronzoni et al., 2016). In that study, a patient carrying a small 6q25 deletion containing only *BAF250b* exhibited the typical features of the syndrome including developmental delay, speech impairment, and agenesis of the corpus callosum (Hoyer et al., 2012; Mignot et al., 2016; Ronzoni et al., 2016).

### Nicolaides-baraitser syndrome (NCBRS)

NCBRS (OMIM #601358) is a disorder that shares remarkable clinical features with CSS. The syndrome is characterized by ID with impaired speech, sparse scalp hair, prominence of the interphalangeal joints and distal phalanges due to decreased subcutaneous fat, characteristic coarse facial features, microcephaly, and seizures (Sousa et al., 2009; Van Houdt et al., 2012). The hallmark differences between NCBRS and CSS come from features of the hands and feet. The representative NCBRS patients show prominent interphalangeal joints and distal phalanges, whereas those with CSS present hypoplasia/aplasia of the fifth fingernails with or without involvement of the terminal phalanges (Wieczorek et al., 2013).

Recently, exome sequencing studies have suggested that NCBRS is associated with *BRM* (*SMARCA2*) mutation. Among the 70 mutations reported in NCBRS patients, one is an in-frame 6-bp deletion, two are in-frame multi-exon deletions affecting the ATPase domain, and 62 are missense mutations. Of the 62 missense mutations, 60 are mapped to the ATPase domain, one is proximal but outside the ATPase domain, and the other is in the bromo domain (Van Houdt et al., 2012; Sousa et al., 2014; Ejaz et al., 2016). Notably, deletions encompassing human *BRM* locus have not been found in NCBRS (Christ et al., 1999). Because null *BRM* KO mice do not show major developmental defects (Koga et al., 2009; Magnani and Cabot, 2009) and none of the identified mutations cause truncated proteins, it has been proposed that the mutations in *BRM* cause GOF or dominant-negative effects. Hence, these mutations possibly interfere with the ability of ATP hydrolysis by native BRM protein (Van Houdt et al., 2012; Ejaz et al., 2016). In addition to *BRM*, missense mutations in *BAF47* (p.Arg366Cys) was also reported to present with NCBRS (Wieczorek et al., 2013).

### Kleefstra's syndrome spectrum (KSS)

Kleefstra's syndrome (OMIM #610253) was known as a recognizable mental retardation syndrome caused by heterozygous mutation in the euchromatin histone methyl transferase 1 (EHMT1) gene (Kleefstra et al., 2006, 2009). However, not all the patients presenting core phenotypes of Kleefstra's syndrome carry an EHMT mutation. Indeed, *de novo* mutations in four epigenetic regulator-encoding genes, *MLL3, MBD5, NR113*, and *BAF47* (*SMARCB1*) were also identified from four of nine EHMT1 mutation-negative patients (Kleefstra et al., 2012). These patients exhibit core features of Kleefstra syndrome; nevertheless they have very heterogeneous phenotypes, which are therefore referred to as Kleefstra syndrome spectrum (KSS). Common phenotypic features among these patients include ID, childhood hypotonia, behavioral anomalies, synophrys, and midface hypoplasia. Investigation with drosophila model revealed direct interactions between MLL3, MBD5, NR113, BAF47 (SMARCB1), and EHMT1 (Kleefstra et al., 2012). The findings suggested that mutations in any protein within the complex of MLL3, MBD5, NR113, BAF47, and EHMT1 could lead to KSS.

### Autism spectrum disorder (ASD)

Autism is a neurodevelopmental disorder characterized by deficits in social skills, speech problems, and restricted and repetitive behaviors. It is well established that a combination of genetic and environmental factors cause autism (Chaste and Leboyer, 2012). However, the underlying genetic basis is unclear for at least 70% of cases. Recent exome sequencing analyses of autism patients have revealed mutations in the following BAF complex components: *BAF155, BAF170, BAF180*, and *BAF250b* (Neale et al., 2012; O'Roak et al., 2012). A *de novo* mutation of genes encoding for the above BAF subunits were identified in patients who showed severe social communication problems with low verbal intelligence quotient (IQ) and nonverbal IQ (Neale et al., 2012; O'Roak et al., 2012).

Recently, mutations in *BCL11A* that encodes BAF100a subunit were also linked to autism. Three patients with microdeletions of 2p15–p16.1 who presented with developmental delay also showed common features of ASD, including moderate to severe ID, facial dysmorphism, and hypotonia (Basak et al., 2015). Although the 2p15–p16.1 microdeletion encompasses ~15 protein-coding genes and six pseudogenes, *BCL11A* is the only common candidate gene in three reported patients with ASD (Basak et al., 2015).

### Schizophrenia

Schizophrenia (SZ; OMIM #181500) is a chronic, severe, and disabling brain disorder that affects ~0.3–0.7% of the world's population. It is characterized by delusion, thought disorder, auditory hallucination, reduced emotional expression and social engagement, disorganized speech, and lacking motivation. The causes of SZ include genetic and/or environmental factors (Zai et al., 2017). In addition to genetic mutations, epigenetic alteration has also been shown to be involved in SZ (Tsankova et al., 2007). BRM was associated with SZ in a screening study of Japanese population (Koga et al., 2009). Mechanistically, the risk alleles of intronic polymorphisms (rs3763627 and rs3793490) were correlated with decreased expression of BRM in post-mortem prefrontal cortex (Koga et al., 2009). Another risk allele, a missense polymorphism (rs2296212, D1546E), located in a highly conserved functional region in mammalian species, reduces the nuclear localization of BRM (Koga et al., 2009). BRM knockout mice exhibit impaired social interaction and pre-pulse inhibition, which suggest that the impaired functions of BRM might play a role in SZ. In cultured mouse primary cortical neurons, knockdown of BRM resulted in impaired dendritic spine growth and abnormal morphology, which is considered an intermediate phenotype of SZ (Bellon, 2007; Lewis and Gonzalez-Burgos, 2007).

In addition to BRM, intronic SNPs in *BCL11A* were significantly associated with schizophrenic patients (Basak et al., 2015). Further studies will be necessary to elucidate the influence of the intronic SNPs on *BCL11A* expression and function.

### Hirschsprung's disease (HSCR)

HSCR is a congenital intestinal disorder characterized by defective migration of enteric neural cells. Recently, a *de novo* heterozygous frameshift mutation in exon 20 of *BAF250b* (c.5789delC p.Pro1930Leufs^*^44) was identified in a patient with HSCR symptoms (Takenouchi et al., 2016). The patient also displayed some CSS features, including coarse facial features, impaired development, epilepsy, and hypoplasia of the corpus callosum and cerebellum. In addition, the patient exhibited congenital cataract disorder and defects in enteric nervous system, which are the cardinal pathologic characteristics of HSCR (Takenouchi et al., 2016). Interestingly, studies in drosophila model confirmed that dysregulation of the ARID1 subunit leads to impaired enteric neural cell migration (Eroglu et al., 2014).

## Conclusion and future perspectives

**Figure 3:**
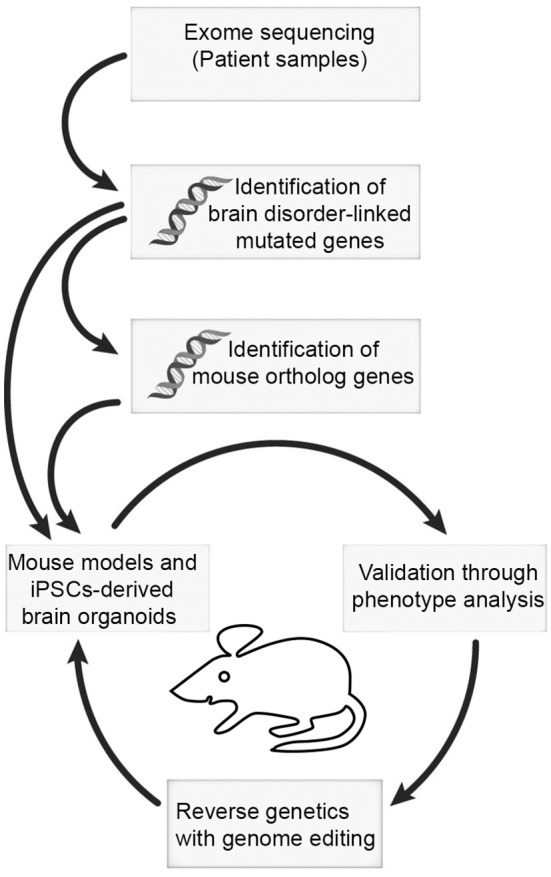
The mouse and iPSC-derived mini-brain organoid pipeline for modeling human brain disorders. Candidates of mutations involved in brain disorders are identified using exome sequencing with DNA samples from patients. When mouse orthologs of candidate genes are identified, they can be edited. Subsequently, phenotypic studies are used to assess validity of the model. Upon validation, reverse genetics approach with genome editing will be applied to restore phenotypes.

It is becoming clear that the BAF complex plays an essential role in mammalian neural development through assembly of developmental stage-specific BAF complexes in NSCs, and post-mitotic neural cells. Therefore, it is not surprising that mutations in BAF subunits are frequently observed in neurological disorders. These disorders are often caused by mutations in many genes encoding BAF subunits, which reflect the fact that different BAF subunits function together to regulate chromatin dynamics and gene expression program. Some potential mechanisms underlying the BAF complex mutations, which cause neurodevelopmental disorders such as GOF or dominant-negative effects, have been proposed. Nevertheless, how these mutations lead to specific neurological phenotypes in patients remains largely unknown. Moreover, most patients with BAF subunit mutations present vast variation of neurological impairments, but the underlining pathological mechanisms still remain an open question. The use of cellular models (e.g., human induced pluripotent stem cells [hiPSCs], cultured brain organoids) and animal models, coupled with new methodologies to generate site-specific editing (e.g., the clustered regularly interspaced short palindromic repeats [CRISPR]/CRISPR-associated protein-9 [Cas9] system) will help to address some of these unsolved questions in the near future (Figure 3). In addition, further studies in structural biology will be necessary to help us understand how the mutations lead to conformational changes of BAF subunits, as well as the deformation of proper functional BAF complexes.

## Author contributions

GS, YX, JR, and TT all contributed to writing and editing the manuscript.

### Conflict of interest statement

The authors declare that the research was conducted in the absence of any commercial or financial relationships that could be construed as a potential conflict of interest. The reviewer IP and handling Editor declared their shared affiliation, and the handling Editor states that the process nevertheless met the standards of a fair and objective review.
